# Actual Scope of Nursing Practice and the Influencing Factors: A Single‐Center Cross‐Sectional Study in China

**DOI:** 10.1155/jonm/1254153

**Published:** 2026-05-31

**Authors:** Wenqian Wang, Ruonan Yang, Xiaomei Liu, Xiaofan Shi, Ziyi Shen, Shan Li, Zhaoying Zhu, Menghan Zhu, Siying Ji, Xinyue Shi, Yunying Hou

**Affiliations:** ^1^ First Affiliated Hospital of Soochow University, Suzhou, Jiangsu, 215006, China, suda.edu.cn; ^2^ School of Nursing, Suzhou Medical College, Soochow University, Suzhou, Jiangsu, 215006, China, scu.edu.tw; ^3^ Suzhou Kowloon Hospital, Shanghai Jiao Tong University School of Medicine, Suzhou, Jiangsu, 215028, China, shsmu.edu.cn

**Keywords:** actual scope of nursing practice, cross-sectional study, influencing factors, nurses

## Abstract

**Aims:**

The “Actual Scope of Nursing Practice” (ASCOP) reflects what nurses are authorized and competent to do and what they do in routine practice. This study examined ASCOP levels and determinants in a tertiary hospital in Suzhou, China, to inform nursing management and hospital administration.

**Design:**

This cross‐sectional study followed the STROBE standards.

**Methods:**

A random sample of 375 nurses was recruited from a Suzhou tertiary hospital between September and October 2025. We collected data using a demographic questionnaire, the ASCOP questionnaire, the growth need strength (GNS) subscale, the job content questionnaire, and the role stressors scale. Descriptive analyses were performed for each variable. Spearman’s rank correlation analyses analyzed key variables, followed by univariate and regression analyses to identify factors associated with the ASCOP.

**Results:**

The mean ASCOP score was 4.03. The final regression analysis explained 38.3% of ASCOP score variance (adjusted *R* ^2^ = 38.3%). Professional title was the strongest positive predictor (*β* = 0.296). Role stressors (*β* = 0.294), psychological demands (*β* = 0.257), and GNS (*β* = 0.234) were also positively associated with ASCOP. Department was statistically associated with ASCOP; however, the effects were modest.

**Conclusion:**

Professional title was the strongest positive predictor of nurses’ ASCOP, suggesting that organizational hierarchy may serve as a key structural influence. This study found a pattern of challenge‐related activities in general nursing units. High psychological demands and moderate role ambiguity were not associated with limiting practice expansion in this single‐center study. Together with the GNS, they were associated with a broader ASCOP.

**Implications for Nursing Management:**

This study advises nursing managers to (1) implement tiered authorization with a clear, graded ASCOP list aligning decision rights and roles to nurse competency; (2) introduce structured bounded autonomy by defining clinical safety thresholds while preserving limited discretion for highly competent nurses; and (3) provide development opportunities to motivated nurses and prioritize workflow support for high‐demand roles.

## 1. Introduction

In line with China’s nursing career development goals [1], the nursing workforce is projected to reach 5.5 million, with a registered nurse density of 3.8 per 1000 people, which is below the WHO benchmark of 5 per 1000. The Healthy China 2030 blueprint projects approximately 4.7 registered nurses per 1000 people, implying that nearly two million additional nurses may be needed to close this gap [2, 3]. Compounded by high turnover [4], these constraints require nursing management to address not only staffing numbers but also the effective deployment of nurses’ professional practice capabilities to safeguard care quality and patient safety [5, 6].

Evidence indicates that when nurses are either required to perform tasks beyond their qualifications or experience (overutilization) or are not provided with sufficient opportunities to fully apply their competencies (underutilization), care effectiveness and work outcomes may deteriorate, and job satisfaction may decline [7, 8]. As care becomes more complex under workforce constraints, conventional managerial approaches such as task assignment and directive supervision may be insufficient to ensure that nurses’ professional roles are enacted in daily workflows [9]. The Actual Scope of Nursing Practice (ASCOP) framework addresses this misalignment by conceptualizing the gap between what nurses are authorized to do and what they actually enact in daily practice [10, 11].

A multinational survey reported that 35%–62% of nurses’ working time was spent on tasks unrelated to direct professional nursing care—an issue described as more pronounced in developing countries—and that administrative and documentation work further constrains professional practice [12, 13]. Such a diversion from essential patient care may disrupt care continuity and compromise patient outcomes [14], underscoring the need for a standardized ASCOP assessment. The ASCOP questionnaire, developed by D’Amour et al., has been widely translated and used internationally [15], and a validated Chinese version was established by Dai et al., enabling standardized ASCOP research in China [16].

This study was guided by the ASCOP theoretical model developed by Déry et al. [17], which conceptualizes the enacted scope of practice as the product of role enactment and the application of professional knowledge within constraints set by legislation, organizational context, and individual competence [18]. The model identifies two core categories of determinants: work characteristics and individual characteristics. Work characteristics include structural and contextual factors such as psychological demands (workload intensity, time pressure, and cognitive effort) and role stressors (role ambiguity, role conflict, and role overload), which shape the organizational conditions under which nurses perform their roles. Individual characteristics encompass professional resources (age, experience, and education) and developmental orientation (growth need strength [GNS]), which influence nurses’ capacity and motivation to expand their enacted practice.

**FIGURE 1:**
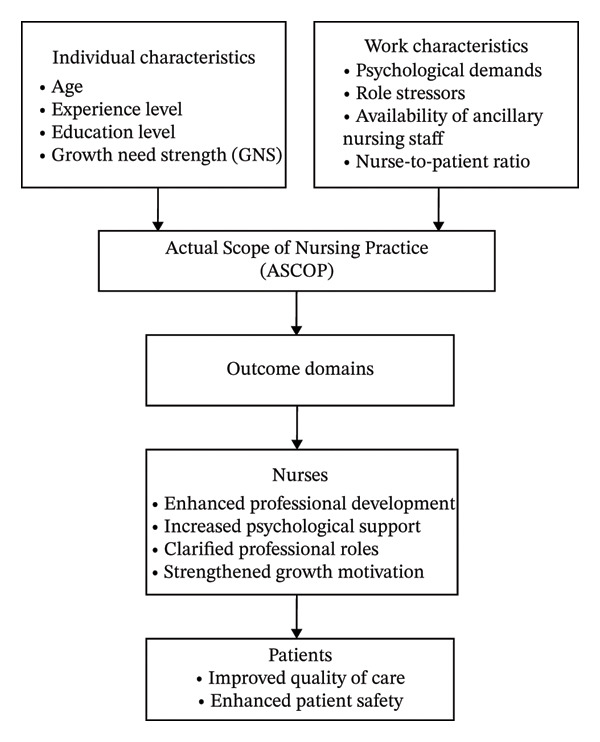
Conceptual framework adapted from the ASCOP model.

Work characteristics present distinct challenges in Chinese hospitals. High documentation burden, frequent workflow interruptions, and task fragmentation intensify psychological demands [19]. Unclear role boundaries and the routine assignments of non‐nursing duties, including patient transport and supply management, may generate role stressors [20]. Simultaneously, variations in nurses’ professional resources and growth orientation (age, experience, education level, and GNS) [21, 22] may also influence role enactment and, thus, ASCOP. The staffing structure, particularly the nurse‐to‐patient ratio and availability of ancillary nursing staff, is a modifiable management lever. International evidence suggests that the nurse‐to‐patient ratio is associated with care delivery and quality and may be relevant to ASCOP [23]. Time–motion studies have further indicated that a meaningful proportion of nurses’ time is spent on delegable tasks, underscoring the role of ancillary nursing staff in protecting time for direct patient care [24]. These findings suggest that the staffing structure may influence ASCOP through task delegation and the time available for professional nursing activities. Building on the ASCOP model, we incorporate a staffing structure as a context‐specific work characteristic. Despite growing international ASCOP research, evidence in China remains limited and is largely derived from single‐department studies [25], constraining generalizability to general hospitals where workflows, case‐mix, and task allocation vary substantially across departments. The associations between the staffing structure (nurse‐to‐patient ratio and ancillary nursing staff availability) and ASCOP have not been systematically examined. Furthermore, no studies have tested whether the ASCOP model’s predicted relationships among work characteristics, individual characteristics, and enacted practice hold in Chinese organizational contexts characterized by hierarchical decision‐making and physician‐led task allocation [26, 27]. Figure 1 presents this study’s conceptual framework.

This study (1) assessed the overall level of ASCOP among nurses in a tertiary hospital in Suzhou, China; (2) compared ASCOP across departments; and (3) explored the model‐informed factors associated with ASCOP, including work characteristics (psychological demands, role stressors, and staffing structure, as indicated by the nurse‐to‐patient ratio and ancillary nursing staff availability) and individual characteristics (age, experience, education level, and GNS). Based on these premises, we hypothesized the following:

• H1: A more favorable nurse‐to‐patient ratio is positively associated with ASCOP.
• H2: Greater availability of ancillary nursing staff is positively associated with ASCOP.
• H3: Greater role stressors are negatively associated with ASCOP.
• H4: Higher psychological demands are negatively associated with ASCOP.
• H5: Higher education level, greater age or more years of experience, and stronger GNS are positively associated with ASCOP.

## 2. Materials and Methods

### 2.1. Study Design

This cross‐sectional study followed the STROBE guidelines to examine the associations between nurses’ individual characteristics, work characteristics, and ASCOP.

### 2.2. Setting and Data Collection

This study was conducted in 21 inpatient departments and three intensive care units (ICUs) of a tertiary hospital in Suzhou, China. Data were collected using an online questionnaire survey. Random sampling was used to recruit clinically active nurses. The survey was administered online via “Wenjuanxing” (Questionnaire Star). Prior to analysis, we screened the submitted questionnaires for data quality. Responses were considered invalid and excluded if they met any prespecified criteria, such as (1) an implausibly short completion time (< 3 min); (2) straight‐lining patterns (selecting the same response option across all scale items), or (3) obviously inconsistent or illogical responses on key items (e.g., contradictory demographic information). Only the questionnaires that passed the quality check were included in the final analysis.

### 2.3. Sample

This study comprised 22 variables, including 15 demographic and individual characteristics, one variable reflecting personal GNS, three variables describing psychological demands, and three variables representing role stress. According to Kendall’s rule of sample size estimation [28], the minimum sample size was estimated to be 110–220. After adding 20% to account for invalid responses, the target sample size was 132–264. In addition, an *a priori* power analysis using G^∗^Power 3.1.9.7 for multiple linear regression analysis (fixed model, *R* ^2^ deviation from zero) with *α* = 0.05, power = 0.80, *f* ^2^ = 0.10, and 22 predictors, indicated a required sample size of 235.

Inclusion criteria were as follows: (1) registered nurses with a valid practicing license who were employed in clinical roles at the study hospital, including staff nurses and first‐line nursing managers (head nurses/ward managers); (2) continuously employed at the study hospital for ≥ 1 year; (3) employed in ward‐based units: internal medicine, surgery, obstetrics and gynecology, pediatrics, or the ICU; and (4) able to provide written informed consent and complete the questionnaire independently. The exclusion criteria were as follows: (1) off‐duty during data collection due to sick leave, maternity leave, or external training; (2) interns, trainees, or visiting nurses from other institutions; (3) working full‐time in nonclinical administrative posts without routine clinical rosters; and (4) emergency, operating room (including postanesthesia care), or other specialized units where ward‐level structural metrics were not comparable.

### 2.4. Variables and Instruments

#### 2.4.1. Demographic Questionnaire

This questionnaire collected data on sex, age, marital status, first and highest academic degrees, hospital of employment, department, professional title, position, type of employment, years of work experience, night‐shift status, annual income, nurse‐to‐patient ratio in the ward, and the number of ancillary nursing staff.

#### 2.4.2. ASCOP Questionnaire

Developed by D’Amour et al. [15], this questionnaire comprises 26 items across six domains. A six‐point Likert scale was used, with higher scores indicating more frequent nursing practice activities and greater ASCOP. The original scale exhibited a Cronbach’s *α* = 0.89, with dimension‐level *α* coefficients ranging from 0.61–0.70. The Chinese version, translated and adapted by Dai Min et al. [16], yielded a total Cronbach’s *α* of 0.908 and dimension‐level *α* coefficients ranging from 0.826–0.915, indicating high internal consistency. This study reported a Cronbach’s *α* of 0.90 and dimension‐level *α* coefficients ranging from 0.87–0.93, demonstrating excellent reliability.

#### 2.4.3. GNS Subscale

GNS reflects an individual’s desire for self‐fulfillment, continuous learning, and personal advancement in their job. It was measured using the GNS subscale originally developed by Hackman and Oldham [29]. The scale comprises six items rated on a five‐point Likert scale (1 = *strongly do not want*, 5 = *strongly want*), with higher mean scores indicating stronger perceived growth needs. In a previous study involving Chinese healthcare professionals [30], the scale demonstrated good validity, with a content validity index (CVI) of 0.87. In this study, the scale was translated into Chinese and then back‐translated into English by an independent bilingual specialist, following a translation–back‐translation procedure. The CVI was 0.83.

#### 2.4.4. Job Content Questionnaire (JCQ‐22)

Psychological demands reflect the perceived intensity of work requirements, such as workload, time pressure, fast work pace, and sustained cognitive and emotional effort. The Chinese version of the JCQ‐22 adapted by Li et al. [31] was used. The JCQ‐22 comprises three main domains with a total of 22 items. The questionnaire was rated on a four‐point Likert scale from 1 (*strongly disagree*) to 4 (*strongly agree*), with higher scores indicating higher levels of the assessed domain. In the present sample, internal consistency was satisfactory, with Cronbach’s *α* = 0.90.

#### 2.4.5. Role Stressors Scale (RSS)

Role stressors refer to stress arising from unclear responsibilities or inconsistent expectations (role ambiguity and role conflict), as well as excessive demands that exceed an individual’s time, energy, or capacity (role overload). The RSS developed by Peterson et al. [32] was used to assess role stressors. The RSS comprises 13 items across three dimensions and uses a five‐point Likert scale, with higher scores indicating greater role stress. The Chinese version adapted by Li and Zhang [33] has demonstrated acceptable internal consistency, with Cronbach’s *α* ranging from 0.74–0.90. In this study, the scale demonstrated good internal consistency, with a Cronbach’s *α* of 0.84.

### 2.5. Data Analysis

SPSS 26.0 was used for analyses, reporting categorical variables as frequencies and percentages, and continuous variables as mean ± SD. Before regression analysis, the assumptions of normality, multicollinearity, and homoscedasticity were examined. Group differences were assessed using *t*‐tests or one‐way analysis of variance. Pearson’s correlation analysis was used for normally distributed continuous data, whereas Spearman’s rank correlation was applied to non‐normal or ordinal data. The Shapiro–Wilk test assessed normality before analysis. In this study, Spearman’s rank correlation analysis was used to examine the associations between ASCOP and psychological demands, role ambiguity, role conflict, role overload, GNS, ancillary nursing staff availability, and nurse‐to‐patient ratio. Prior to multiple linear regression analysis, assumptions including normality of residuals, multicollinearity, and homoscedasticity were evaluated. Variables that were significant in the univariate analyses (*p* < 0.05) were entered into the multiple linear regression model to identify the determinants of ASCOP scores. The variables that did not meet these criteria were excluded. A two‐tailed significance level of *α* = 0.05 was applied to all analyses.

## 3. Results

A total of 391 questionnaires were received, of which 16 were excluded because they were invalid or did not meet the inclusion criteria. A final total of 375 valid responses corresponded to a return rate of 95.9%. All the included questionnaires were completed without missing data.

### 3.1. Demographic Characteristics of Participants

Among the 375 nurses included, 355 were women (94.67%) with an average age of 29.86 ± 6.85 years. More than half (52.27%) were unmarried. Most nurses started with associate degrees (72.27%) or bachelor’s degrees (22.93%), though by the time of the survey, nearly 79.20% had upgraded to bachelor’s degrees. Only four nurses (1.07%) held master’s degrees or higher. Most respondents worked in internal medicine (53.60%), about 24.27% in surgery, and 15.47% in ICUs. Smaller numbers worked in pediatrics (3.47%) and obstetrics and gynecology (3.20%). Most nurses (80.00%) were on long‐term contracts, while 14.13% had temporary contracts and 2.40% held establishment positions, and 3.47% were in other categories. Regarding work experience, 48.27% had 1–5 years of service, 23.20% had 6–10 years, 12.80% had 11–15 years, and 15.73% had been working for over 15 years. Professional titles ranged from staff nurse (30.90%) and senior nurse (33.60%) to nurse‐in‐charge (26.70%) and associate chief nurse or above (8.80%). Despite these varied titles, most nurses (83.20%) worked in staff nurse positions, with only 8.80% as team leaders and 8.00% as unit managers or above. A large proportion of participants (89.87%) worked night shifts, and two‐thirds (67.47%) reported a monthly income between 5001 and 10,000 RMB. The average nurse‐to‐patient ratio was 0.63 ± 0.56; each ward employed a mean of 5.43 ± 5.42 ancillary nursing staff.

### 3.2. Descriptive Analysis of ASCOP

Table 1 presents the mean scores for the primary study variables. The overall mean score on the ASCOP scale was 4.03 ± 1.16. Among the six dimensions, Patient and Family Education ranked highest (4.44 ± 1.30), followed by Communication and Care Coordination (4.37 ± 1.29). The mean score was lowest in Integration and Management of Members (3.08 ± 1.56). Other dimensions, including Care Quality and Patient Safety (4.06 ± 1.40), Assessment and Care Planning (3.91 ± 1.35), and Knowledge Update and Utilization (4.16 ± 1.37), showed moderate levels of practice engagement.

**TABLE 1:** Descriptive analysis of ASCOP (*n* = 375).

Variables	Mean	SD	Minimum	Maximum
ASCOP	4.03	1.16	1	6
Integration and management of members	3.08	1.56	1	6
Care quality and patient safety	4.06	1.40	1	6
Assessment and care planning	3.91	1.35	1	6
Communication and care coordination	4.37	1.29	1	6
Patient and family education	4.44	1.30	1	6
Knowledge update and utilization	4.16	1.37	1	6

### 3.3. Univariate Analysis of Categorical Factors Associated With ASCOP

The results of the univariate analysis examining categorical variables in relation to ASCOP are summarized in Table 2. No significant differences were observed between male and female nurses (*Z* = −0.473, *p* = 0.636). Age had a significant effect (*H* = 15.681, *p* < 0.001), with nurses aged ≥ 45 years showing the highest scores (4.78 ± 0.89), followed by those aged 31–45 years (4.29 ± 1.05), and those aged ≤ 30 years (3.87 ± 1.20). Night‐shift status was significantly associated with ASCOP (*Z* = −4.005, *p* < 0.001); nurses not working night shifts scored higher (4.74 ± 0.96) than those who did (3.95 ± 1.16). Marital status showed significant differences (*Z* = −2.904, *p* = 0.036), with married nurses scoring higher (4.21 ± 1.10) than unmarried nurses (3.87 ± 1.20). The first academic credentials obtained were significant (*H* = 9.368, *p* = 0.025), indicating that nurses with bachelor’s degrees or above had higher scores, whereas the highest academic credentials were not significant (*p* = 0.377). Significant differences were found across departments (*H* = 11.950, *p* = 0.018), with nurses in obstetrics and gynecology scoring the highest (4.40 ± 1.17), followed by those in surgery (4.28 ± 1.11) and internal medicine (3.99 ± 1.19). Employment type showed no significant difference (*p* = 0.139). Years of nursing experience had a strong effect (*H* = 31.882, *p* < 0.001), with those working more than 15 years achieving the highest scores (4.74 ± 1.01). Professional title was also a significant factor (*H* = 37.163, *p* < 0.001); ASCOP scores increased with higher titles, with associate chief nurse or above scoring the highest (5.00 ± 0.96). Significant differences were observed across position categories (*H* = 22.262, *p* < 0.001), with scores increasing progressively from staff nurses (3.91 ± 1.15) to team leaders (4.44 ± 1.01) to unit managers or above (4.81 ± 1.05). Monthly income was significantly associated with ASCOP (*H* = 10.524, *p* = 0.015), with those earning more than 10,000 CNY achieving the highest mean scores (4.58 ± 0.96).

**TABLE 2:** Univariate analysis of categorical factors associated with ASCOP (*n* = 375).

Parameter	*N*	%	Score (mean ± SD)	*Z/H*	*p*
Gender				−0.473	0.636
Male	20	5.33	3.96 ± 1.20		
Female	355	94.67	4.03 ± 1.16		
Age (year)					
≤ 30	244	65.07	3.87 ± 1.20	15.681	< 0.001
31–45	122	32.53	4.29 ± 1.05		
≥ 45	9	2.40	4.78 ± 0.89		
Night shift				−4.005	< 0.001
Yes	337	89.87	3.95 ± 1.16		
No	38	10.13	4.74 ± 0.96		
Marital status				−2.904	0.036
Unmarried	196	52.27	3.87 ± 1.20		
Married	179	47.73	4.21 ± 1.10		
First obtained academic credential				9.368	0.025
Secondary specialized school	15	4.00	4.92 ± 0.30		
Associate degree	271	72.27	3.96 ± 1.07		
Bachelor’s degree	86	22.93	4.10 ± 1.12		
Master’s degree or above	3	0.80	3.30 ± 1.29		
Highest obtained academic credential				1.951	0.377
Associate degree	74	19.73	3.88 ± 1.26		
Bachelor’s degree	297	79.20	4.07 ± 1.14		
Master’s degree or above	4	1.07	3.91 ± 0.68		
Department				11.950	0.018
Internal medicine	201	53.60	3.99 ± 1.19		
Surgery	91	24.27	4.28 ± 1.11		
ICU	58	15.47	3.70 ± 1.04		
Pediatrics	13	3.47	3.97 ± 1.18		
Obstetrics and gynecology	12	3.20	4.40 ± 1.17		
Nature of employment				5.499	0.139
Establishment	9	2.40	4.45 ± 1.14		
Long‐term contract	300	80.00	4.05 ± 1.17		
Temporary contract	53	14.13	3.97 ± 1.15		
Other	13	3.47	3.33 ± 0.76		
Years of nursing experience				31.882	< 0.001
1–5 years	181	48.27	3.78 ± 1.25		
6–10 years	87	23.20	3.99 ± 1.00		
11–15 years	48	12.80	4.14 ± 0.90		
> 15 years	59	15.73	4.74 ± 1.01		
Professional title				37.163	< 0.001
Staff nurse	116	30.90	3.73 ± 1.23		
Senior nurse	126	33.60	3.87 ± 1.14		
Nurse‐in‐charge	100	26.70	4.24 ± 0.97		
Associate chief nurse or above	33	8.80	5.00 ± 0.96		
Position				22.262	< 0.001
Staff nurse	312	83.20	3.91 ± 1.15		
Team leader	33	8.80	4.44 ± 1.01		
Unit managers or above	30	8.00	4.81 ± 1.05		
Monthly income (CNY)				10.524	0.015
< 3000	4	1.07	3.54 ± 1.28		
3000–5000	87	23.20	3.80 ± 1.21		
5001–10,000	253	67.47	4.05 ± 1.15		
> 10,000	31	8.27	4.58 ± 0.96		

*Note:* CNY = Chinese Yuan, 1 Chinese Yuan = 0.71 dollars.

### 3.4. Spearman’s Rank Correlation Analysis of Variables

The results revealed several significant associations between the variables listed in Table 3. ASCOP was moderately and positively correlated with GNS (*r* = 0.403, *p* < 0.01), psychological demands (*r* = 0.539, *p* < 0.01), and role ambiguity (*r* = 0.456, *p* < 0.01), whereas role conflict (*r* = 0.141, *p* < 0.01) showed a small positive association with ASCOP. In contrast, ASCOP was not significantly correlated with the nurse‐to‐patient ratio, ancillary nursing staff, or role overload (*p* > 0.05). Among the independent variables, GNS was positively related to psychological demands (*r* = 0.601, *p* < 0.01), role ambiguity (*r* = 0.392, *p* < 0.01), and role conflict (*r* = 0.336, *p* < 0.01). Similarly, psychological demands were strongly correlated with role ambiguity (*r* = 0.726, *p* < 0.01). Role conflict was also moderately correlated with role overload (*r* = 0.378, *p* < 0.01). No significant correlation was observed between role overload and ambiguity (*r* = 0.004, *p* > 0.05).

**TABLE 3:** Spearman’s rank correlation analysis of variables (*n* = 375).

Variable	1	2	3	4	5	6	7	8
(1) ASCOP	—							
(2) Nurse‐to‐patient ratio	−0.103	—						
(3) Ancillary nursing staff	−0.058	0.132^∗^	—					
(4) GNS	0.403^∗∗^	−0.107^∗^	0.057	—				
(5) Psychological demands	0.539^∗∗^	−0.075	−0.022	0.601^∗∗^	—			
(6) Role overload	0.067	−0.129^∗^	−0.070	0.114^∗^	−0.070	—		
(7) Role ambiguity	0.456^∗∗^	−0.129^∗^	−0.052	0.392^∗∗^	0.726^∗∗^	0.004	—	
(8) Role conflict	0.141^∗∗^	−0.125	−0.008	0.336^∗∗^	0.161^∗∗^	0.378^∗∗^	0.179^∗∗^	—

*Note:* ASCOP: Actual Scope of Nursing Practice.

Abbreviation: GNS, growth need strength.

^∗^Significant at 0.05.

^∗∗^Significant at 0.01.

### 3.5. Multiple Linear Regression Model of Factors Associated With ASCOP

Multiple linear regression analysis was used to determine the variables affecting ASCOP, as shown in Table 4. A stepwise multiple linear regression analysis was conducted to identify the most parsimonious set of predictors for the overall ASCOP score. The final model retained professional title, department, role stressors, psychological demands, and GNS as significant predictors of ASCOP performance. Among the continuous variables, role stressors (*β* = 0.294, *B* = 1.318, 95% CI 0.869–1.881, *p* < 0.001) showed the strongest association with ASCOP, followed by psychological demands (*β* = 0.257, *B* = 0.025, 95% CI 0.011–0.039, *p* < 0.001) and GNS (*β* = 0.234, *B* = 0.033, 95% CI 0.018–0.048, *p* < 0.001). Compared with staff nurses, nurses with higher professional titles reported significantly higher ASCOP scores, including senior nurses (*β* = 0.105, *B* = 0.259, 95% CI 0.026–0.492, *p* = 0.029), nurse‐in‐charge (*β* = 0.204, *B* = 0.537, 95% CI 0.286–0.787, *p* < 0.001), and associate chief nurse or above (*β* = 0.296, *B* = 1.232, 95% CI 0.868–1.597, *p* < 0.001). In addition, relative to internal medicine, nurses working in surgery (*β* = 0.099, *B* = 0.269, 95% CI 0.049–0.488, *p* = 0.017) and obstetrics and gynecology (*β* = 0.088, *B* = 0.579, 95% CI 0.034–1.113, *p* = 0.034) reported higher ASCOP scores. The final regression model was statistically significant, *F* (8, 366) = 30.00, *p* < 0.001, and explained 39.6% of the variance in ASCOP scores (*R* ^2^ = 0.396, adjusted *R* ^2^ = 0.383).

**TABLE 4:** Multiple linear regression models of factors associated with ASCOP (*n* = 375).

Parameter	*β*	SE	*B*	*t*	*p*	95% CI	
Constant		0.371	−0.960	−2.590	0.010	−1.688	−0.231
Professional title (reference: staff nurse)							
Senior nurse	0.105	0.118	0.259	2.264	0.029	0.026	0.492
Nurse‐in‐charge	0.204	0.128	0.537	4.207	< 0.001	0.286	0.787
Associate chief nurse or above	0.296	0.185	1.232	6.651	< 0.001	0.868	1.597
Department (reference: internal medicine)							
Surgery	0.099	0.112	0.269	2.406	0.017^∗^	0.049	0.488
Obstetrics and gynecology	0.088	0.271	0.579	2.133	0.034^∗^	0.034	1.113
Role stressors	0.294	0.222	1.318	5.945	< 0.001	0.869	1.881
GNS	0.234	0.008	0.033	4.303	< 0.001	0.018	0.048
Psychological demands	0.257	0.007	0.025	3.589	< 0.001	0.011	0.039

*Note:* ASCOP: Actual Scope of Nursing Practice; *β*: the standardized coefficient; B: the unstandardized coefficient. The dependent variable was the overall ASCOP score.

Abbreviations: CI, confidence interval; GNS, growth need strength; SE, standard error.

^∗^Significant at 0.05.

## 4. Discussion

Assessing the extent to which ASCOP is enacted in clinical practice helps to clarify how nurses’ potential capabilities are translated into routine care activities and what structural conditions may constrain this translation. In this study, the mean overall ASCOP was 4.03 out of 6, indicating a moderate level of enacted practice. This score exceeds the means reported in Canadian studies (3.21 by Déry et al. [7] and 3.47 by D’Amour et al. [15]) but falls short of the scores reported in Lebanon (4.42) and Saudi Arabia (4.46) [34, 35]. These cross‐national differences likely reflect variations in nurse autonomy across health systems and organizational contexts, the structuring of interprofessional roles and responsibilities, and resource availability. In Chinese hospitals, hierarchical decision‐making structures, physician‐led task allocation, and persistently high workloads are well‐documented constraints that may limit nurses’ opportunities to enact the full scope of practice [26, 27]. These organizational and role‐related constraints may act as structural barriers to translating professional capabilities into routine care.

The ASCOP domain scores revealed meaningful variations in the enacted nursing roles across practice areas. “Patient and Family Education” (*M* = 4.44) and “Communication and Care Coordination” (*M* = 4.37) were the highest‐scoring domains, which is consistent with previous studies [35, 36]. This pattern likely reflects the high degree of standardization in current hospital management systems. Tasks such as health education and interprofessional communication are deeply embedded in core nursing responsibilities and quality appraisal frameworks, making their enactment routine [37, 38]. In contrast, “Assessment and Care Planning” (*M* = 3.91), “Knowledge Update and Utilization” (*M* = 4.16), and “Care Quality and Patient Safety” (*M* = 4.06) scored moderately to highly. This finding suggests that nurses possess the foundational capacity for knowledge updating and evidence use. However, when comprehensive clinical pathways and standardized assessment tools are lacking, evidence may not be consistently embedded stably at key clinical decision points [39], potentially compromising the consistency and routinization of quality and safety practices in daily work. Consistent with prior research [15, 35, 36, 40], “Integration and Management of Members” was the lowest scoring and most variable domain (*M* = 3.08, SD = 1.56), suggesting that leadership and team‐development activities are unevenly enacted rather than routinized. This domain encompasses higher complexity leadership activities, such as precepting and supervising new staff and students, identifying team learning needs, and organizing training. These activities often depend on discretionary authority, experience, and time resources, which may reflect the hierarchical features of authorization and task allocation arrangements. When preceptorship and team supervision are not operationalized as assessable role expectations or formalized role arrangements within clinical units, they are more likely to be implicitly treated as responsibilities and decision rights aligned with higher professional ranks, becoming concentrated within a smaller subset of staff. Under compounded pressures of staffing shortages and high clinical workload, preceptorship and team‐development activities are readily displaced as “add‐on” work [41–43], limiting their routinization and standardization across the nursing workforce. Therefore, practical strategies for nurse managers may include the following: (1) improving the consistency with which preceptorship and team‐development responsibilities are specified and enacted across role levels, supported by clear boundaries of authority; (2) allocating protected time and measurable workload credits for supervision, coaching, and training activities within staffing models and scheduling [44]; and (3) establishing competency‐based preparation and evaluation for supervisors, supported by standardized tools to identify learning needs and track training delivery.

In the final regression model, ASCOP scores were significantly associated with demographic factors (department and professional title), individual characteristics (GNS), and work characteristics (role stressors and psychological demands). These findings are discussed, along with the correlational results. Contrary to H1 and H2, neither the nurse‐to‐patient ratio nor the ancillary nursing staff was positively correlated with ASCOP; therefore, neither was retained in the final model. This null finding may reflect a level‐of‐analysis mismatch, as staffing indicators are measured at the unit or hospital level, whereas ASCOP captures individual‐level enacted practices. This also suggests that staffing increases without an accompanying workflow redesign may improve work comfort but are unlikely to shift practice boundaries [45].

This study found that, compared with staff nurses, nurses with higher professional titles reported higher levels of ASCOP, with the strongest association observed for associate chief nurse and above (*β* = 0.296, *p* < 0.001). This finding aligns with that of Déry et al. [7], who suggested that the link between role title and enacted scope of practice reflects title‐linked differences in authorization, responsibility allocation, and access to practice opportunities embedded in organizational role structures. Drawing on Kanter’s theory of structural empowerment [46], higher titles may confer preferential access to information, resources, and supportive networks, thereby enabling participation in a wider range of practical activities. Within prevailing healthcare evaluation and promotion systems, advancement is typically accompanied by heightened expectations for leadership, teaching, and quality management. Higher titled nurses may, therefore, be entrusted with greater decision latitude and more central roles in interprofessional collaboration, complex case guidance, and clinical process improvement, thereby expanding their enacted ASCOP. However, an overly title‐based hierarchy may rigidify practice boundaries and underutilize capable early career nurses, underscoring the need to complement title‐based allocations with competency‐based flexible authorization mechanisms.

In addition, departmental context was statistically associated with nurses’ ASCOP scores. Compared with nurses in internal medicine, nurses in surgery (*β* = 0.099, *p* < 0.05) and obstetrics and gynecology (*β* = 0.088, *p* < 0.05) reported significant but relatively modest advantages in ASCOP. Prior studies have often attributed specialty differences to variations in workload [47, 48]. However, our findings suggest that cross‐departmental differences in ASCOP are not only workload‐related but may also partly reflect how clinical task characteristics reshape nurses’ ASCOP. First, compared to the more predictable routines of internal medicine, perioperative and intrapartum care involve greater urgency and uncertainty, which may prompt greater reliance on real‐time assessment and protocol‐guided rapid response, supporting the adaptive expansion of ASCOP. Second, because the surgical and obstetric pathways are tightly coupled and highly interdependent, nurses may more often assume coordinating roles involving interprofessional communication, information integration, and resource coordination, thereby strengthening the enactment of ASCOP‐related activities. Notably, the effect sizes are significant but modest, suggesting that departmental influences on ASCOP may be limited rather than uniform. These studies [23, 49] offer a plausible explanation, suggesting that highly specialized environments can have a double‐edged effect, enhancing emergency and specialty‐specific capabilities while narrowing practice toward technology‐and procedure‐focused work, which may crowd out the more comprehensive functions captured by ASCOP. This also indicates that, when planning nursing workforce allocation, managers should avoid overstating the natural role expansion of specialty nurses and should proactively use institutional design to reduce potential practice barriers created by specialization. For example, establish cross‐specialty rotation programs to maintain broad clinical competencies and hold quarterly interdisciplinary case conferences where specialty nurses present comprehensive care cases to reinforce holistic practice patterns.

Contrary to H3, role stressors were positively associated with ASCOP in the regression model (*β* = 0.294, *p* < 0.001), rather than showing the hypothesized negative association. Examination of individual dimensions showed that role ambiguity was most strongly correlated with ASCOP (*r* = 0.456, *p* < 0.01), exceeding role conflict (*r* = 0.141, *p* < 0.01) and role overload (*r* = 0.067, *p* > 0.05), suggesting that the observed positive association between role stressors and ASCOP in this study may be primarily related to role ambiguity. This finding is broadly consistent with the results reported by Déry et al. [11, 18] and differs from those reported by Saralegui‐Gainza et al. and the domestic study [23, 35]. One possible explanation is that the meaning and effects of role ambiguity may vary across departmental contexts. Our sample, similar to that of Wrzesniewski and Dutton, primarily comprised nurses working in general inpatient wards. From a job crafting perspective [50], nurses may construe a moderate degree of role ambiguity as a structural void, which may be associated with more proactive role negotiation and task reconfiguration, and may thereby be linked to the adaptive expansion of their professional roles. However, studies have focused on high‐intensity, high‐risk departments, such as ICUs and emergency departments. In such settings, role ambiguity may instead function as a typical hindrance stressor [51]. As unclear role boundaries may disrupt critical workflows and compromise patient safety, these units may require a high degree of structured role clarity to safeguard both practice effectiveness and patient safety [52]. This suggests that nursing managers in similar settings may consider differentiated role boundary management strategies. In general wards, one possible approach is structured bounded autonomy, with clearly defined clinical safety thresholds while preserving limited discretion for highly competent nurses. In high‐risk units (ICUs and emergency departments), a stronger emphasis on role clarity may be warranted through clearer role‐responsibility definitions, simulation‐based workflow reinforcement, and structured handoff processes.

Although H4 anticipated a negative impact, our results aligned with prior research [35], identifying psychological demands as a significant positive predictor of ASCOP (*β* = 0.257, *p* < 0.001). A plausible interpretation is that psychological demands reflect the perceived intensity of work demands, such as workload, time pressure, fast work pace, and sustained cognitive and emotional effort. Under complex and multitasking clinical demands, higher psychological demands within a certain range may be appraised as challenge stress [49, 53]. To meet these demands, nurses may employ proactive coping strategies, such as enhanced clinical assessment, interprofessional coordination, and problem‐solving, which may be associated with a broader ASCOP [54]. As these findings are based on cross‐sectional data, they reflect associations rather than causation. This positive association is therefore conditional: When resources are insufficient or demands accumulate over time, psychological demands may shift from challenges to hindrance stressors, leading to adverse outcomes. Therefore, nursing management should strengthen its resources and support for high‐demand roles. First, the staffing ratios should be adjusted during high‐demand periods by increasing the number of staff per patient. Second, dedicated support staff should be assigned to non‐nursing tasks, and workload monitoring systems should be deployed to trigger resource reallocation. Third, quarterly training on time management, prioritization, and stress management should be provided to prevent demands from becoming unsustainable.

Supporting H5, the results indicated that, alongside external work characteristics, nurses’ GNS was also a significant positive predictor of ASCOP (*β* = 0.234, *p* < 0.001) [35], although the effect was slightly smaller than that of psychological demands. Nurses with higher GNS may appraise complex clinical tasks not simply as resource depletion but as opportunities to gain resources, such as enhanced professional competence and a stronger sense of accomplishment. Consequently, they may be less satisfied with completing prescribed duties alone and more inclined to cross role boundaries by participating in higher skilled nursing activities that support professional development, which may be associated with a broader scope of ASCOP [55]. Notably, the magnitude of this pathway was modest, which may suggest that high growth motivation does not readily translate into expanded practice without structural organizational support. Therefore, nursing management should not only recognize individual differences in GNS but also establish matched developmental pathways [56]. Nurses with high GNS scores implement fast‐track competency assessments, provide early access to complex cases and research projects, and facilitate cross‐unit rotations. Lower GNS nurses consolidate core competencies through structured refreshers and strengthen support through mentorship and regular feedback.

### 4.1. Limitations

This study has several limitations. First, the use of a single‐center convenience sample may limit generalizability, and the observed associations may primarily reflect ASCOP patterns within one institution rather than across diverse hospital types and regions. Second, this study did not consider the impact of the nursing practice environment, which may significantly affect nurses’ role enactment, clinical decision‐making, and practice scope. Future research should incorporate validated instruments that assess environmental factors, such as workload, teamwork, resource adequacy, and managerial support. This will enable a more comprehensive understanding of how contextual factors shape nurses’ ASCOP. Third, the predominance of female participants is another limitation. Sex differences may influence nurses’ practice scope, professional attitudes, and coping strategies, which could affect study outcomes. Future research should ensure a more balanced sex distribution to better represent the overall nursing workforce and to examine potential sex‐related differences in ASCOP scores. Finally, the cross‐sectional, self‐reported design precludes causal inferences and limits the testing of underlying mechanisms. Longitudinal designs and, where appropriate, multilevel models are required to examine how individual‐ and unit‐level factors jointly influence ASCOP.

## 5. Conclusions

This study found that professional title was the strongest predictor of nurses’ ASCOP scores in this single‐center sample, suggesting that practice opportunities and responsibility allocation may be closely linked to institutionalized hierarchies. Most notably, within the nursing units included in this study, psychological demands and role stressors—particularly role ambiguity—did not show the expected inhibitory effects. These results suggest that, in this context, high psychological demands and a certain degree of role ambiguity were not observed to function as barriers to practice and may instead reflect conditions associated with a broader ASCOP. Moreover, although the department was significant, its relatively modest explanatory power suggests that cross‐department differences provide a contextual background for ASCOP, rather than serving as a dominant driver. Overall, in nursing units with similar organizational contexts, management practice may benefit from a shift away from broadly adding resources to targeted structural redesign: Tiered authorization can be implemented by developing an explicit, graded ASCOP list that closely aligns decision rights and role boundaries with nurses’ competency levels, thereby reducing capability‐role misalignment driven by seniority‐based allocation. Structured bounded autonomy may be introduced by clearly defining clinical safety thresholds while deliberately preserving limited discretion for highly competent nurses, which may help reduce overreliance on rigid procedures and encourage proactive job crafting and role expansion. Differentiated support should also be provided by offering development opportunities to nurses with strong growth motivation and prioritizing workflow coordination and support for high‐demand roles, thereby promoting the sustainable expansion of ASCOP.

## Author Contributions

Wenqian Wang: conceptualization, methodology, formal analysis, investigation, data curation, writing–original draft, and writing–review and editing. Ruonan Yang: data curation, formal analysis, and methodology. Xiaomei Liu: investigation and data curation. Xiaofan Shi: investigation. Ziyi Shen: investigation. Shan Li: investigation. Zhaoying Zhu: investigation. Menghan Zhu: investigation. Siying Ji: data curation. Xinyue Shi: investigation.Yunying Hou: writing–review and editing, funding acquisition, supervision, and resources.

## Funding

No funding was received for this manuscript.

## Disclosure

All authors read and approved the final manuscript.

## Ethics Statement

Ethical approval for this study was granted by the Ethics Committee of Suzhou Kowloon Hospital (MS‐2025‐002).

## Consent

All participants were fully informed about the purpose, procedures, and benefits of the research and provided their written informed consent before participation.

## Conflicts of Interest

The authors declare no conflicts of interest.

## Data Availability

The data that support the findings of this study are available from the corresponding author upon reasonable request.
